# The Evolving Treatment Landscape of Medullary Thyroid Cancer

**DOI:** 10.1007/s11864-023-01145-5

**Published:** 2023-11-18

**Authors:** Marta Laganà, Valentina Cremaschi, Andrea Alberti, Danica M. Vodopivec Kuri, Deborah Cosentini, Alfredo Berruti

**Affiliations:** 1 Medical Oncology Unit, Department of Medical and Surgical Specialties, Radiological Sciences and Public Health, University of Brescia at ASST Spedali Civili, 25123 Brescia, Italy; 2 Department of Endocrinology, Diabetes, and Metabolism, University Of Alabama, 619 19Th St S, Birmingham, AL 35249 USA

**Keywords:** Medullary thyroid, Cancer, RET mutations, MultiTKI, RET inhibitors, Metastatic MTC, Future options, Pretreated MTC

## Abstract

Genetic assessment is crucial to address the correct treatment for advanced medullary thyroid cancer (MTC). Multi tyrosine kinase inhibitors (mTKIs) cabozantinib and vandetanib are good first line options, even vandetanib prescription is currently limited to RET mutated patients. Selective RET inhibitors such as pralsetinib could be a preferred upfront treatment in case of RET mutated MTC presenting common or gatekeeper RET mutations (e.g. M918T; V804L/M). Selpercatinib, otherwise, can be prescribed as the second line after disease progression to mTKIs. The best option for subsequent lines is to consider inclusion in clinical trials or alternatively other mTKIs such as sunitinib, sorafenib, lenvatinib, or pazopanib could be evaluated. New perspectives include next-generation RET inhibitors able to overcome resistance mechanisms responsible for disease progression to standard mTKIs and RET inhibitors, and immunotherapy for MTC presenting with high tumor mutational burden.

## Introduction

Medullary thyroid carcinoma (MTC) is a rare neuroendocrine tumor that originates from the parafollicular cells of the thyroid gland and accounts for up to 4% of all thyroid cancers in the West [1, 2, 3••]. MTCs occur either as sporadic tumors or as inherited components of multiple endocrine neoplasia (MEN) type 2. They can secrete calcitonin and are unable to produce calcitrides, along with other humoral substances that may contribute to paraneoplastic syndromes. The primary treatment for MTC is extensive and meticulous surgical resection. External beam radiation therapy (EBRT) has a limited role. For patients with progressive or symptomatic metastatic disease who cannot be treated with surgery, radiotherapy, or other focal ablative interventions, targeted systemic therapies are effective interventions. Between 2011 and 2020, the FDA and EMA approved four tyrosine kinase inhibitors for the treatment of progressive, unresectable, locally invasive, and/or advanced metastatic MTC that is not amenable to other treatments. In this review, we intend to focus on the state of the art of available treatment options for advanced/metastatic MTC in Europe and the USA, as well as future treatments.

### Epidemiology

MTC accounts for 1–2% of all thyroid cancer. Sporadic MTC accounts for about 80% of all cases of the disease and presents in the fifth or sixth decade of life. Inherited forms of the disease tend to present at earlier ages. In contrast with thyroid epithelial cell tumors, the female-to-male ratio for MTC is nearly equal [1].

### Genetics

MTC can be sporadic (somatic mutation, 75% of cases) and hereditary (germline mutation, 25% of cases), with the later form being part of MEN2 syndrome types A and B. MEN2A is the most frequent subtype (95%) and consists of MTC (present 100% of the case), pheochromocytoma (PHEO, up to 50%), primary hyperparathyroidism (PHPT, up to 30%), and with less frequency cutaneous lichen amyloid and Hirschsprung’s disease [1, 2]. MEN2B accounts for only 5% of inherited MTC, and it is the most aggressive form of MEN2 characterized by early onset MTC, PHEO, and a characteristic phenotype: mucosal ganglioneuromas and marfanoid habitus [1, 2]. It is a current standard of care for all patients with newly diagnosed MTC to undergo genetic testing because up to 7% of seemingly sporadic MTC cases are in fact de novo hereditary mutations (meaning not inherited from either parent). In addition, approximately 75% of patients with MEN2B have a de novo germline RET mutation. Children with MEN2 should undergo prophylactic thyroidectomy; however, the timing of surgery depends on the *RET* mutation American Thyroid Association (ATA) risk category [1, 4].

The *RET* oncogene is the most common driver mutation in MTC. It is present in 100% of MEN2 syndromes and about 45% of sporadic MTC. Mutually exclusive *RAS* mutations (*HRAS* and *KRAS*) have been reported in approximately 15% of sporadic MTC, RET, and RAS proto-oncogene mutations are detected in approximately 90% of MTCs and are the predominant drivers of these tumors. In the remainder cases of sporadic MTC do not have an identifiable driver mutation [5]. *RET* is a proto-oncogene that encodes a tyrosine kinase transmembrane receptor. The normal RET receptor activation process starts with the binding of growth factors to a co-receptor, which causes RET dimerization and phosphorylation at the terminal kinase region with resultant activation of the *RAS/MAPK* and *PI3K/AKT* pathway involved in cell proliferation, differentiation, survival, growth, and migration [6–8]. About 95% of mutations in MEN2A take place in the cysteine-rich domain of RET extracellular region (exon 8 to 11) creating disulfide-bonded RET homodimers with subsequent ligand-independent activation of the receptor at the kinase-region [9]. The most frequent and aggressive form of MEN2B is characterized by a mutation in codon M918T of exon 16 which increases ATP binding affinity to RET monomers causing autophosphorylation without the need for receptor dimerization. Generally, closer is the activating mutation to the kinase domain of the RET receptor, and more aggressive is the disease [9]. In sporadic MTC the most common somatic *RET* mutation is M918T, but alterations in other codons (like V804L/M), deletions, and duplications have also been identified [7•]. Since it is unclear how knowledge of a specific somatic (acquired) RET mutation should impact initial clinical management and follow-up, it is not routinely necessary to evaluate primary tumor samples for RET mutational status. However, somatic mutational profiling of tumor tissue should be performed in patients being considered for a systemic therapy. Today, the presence or absence and the type of RET mutations have an impact on the choice of first-line therapy, RET inhibitor drugs, or multi-kinase inhibitors (MKIs).

### Clinical presentation and behavior

MTC has a wide range of clinical behaviors, varying from indolent to aggressive tumors and based on stage, in particular, 5-year relative survival for stage I to III is about 93%, whereas 28% for stage IV [10••]. Sporadic MTC accounts for approximately 75% of all cases. The typical age of presentation is in the fourth and sixth decades of life and most cases is an asymptomatic solitary thyroid nodule, therefore the diagnosis of MTC is often done when the tumor has progressed outside of the thyroid to cervical lymph nodes [11]. Up to 15% have symptoms of upper aerodigestive tract compression or invasion such as dysphagia or hoarseness. Systemic symptoms may occur due to hormonal secretion by the tumor, calcitonin, and calcitonin gene-related peptide, or other substances that can cause diarrhea or facial flushing. In addition, occasional tumors secrete corticotropin (ACTH), causing ectopic Cushing syndrome. In the index case, the clinical presentation and manifestations of MEN2-associated MTC are similar to those of sporadic MTC. The most common presentation is that of a solitary thyroid nodule or cervical lymphadenopathy. Early diagnosis (before any clinical manifestations) by screening of “at-risk” family members in MEN2 kindred is important because MTC is a life-threatening disease that can be cured or prevented by early thyroidectomy. Approximately 5 to 10% have distant metastatic disease [1, 6]. Distant metastases may occur in the liver, lung, bones, and, less often, brain and skin. Nodal metastases are more common in patients with multifocal disease [7•]. However, as calcitonin screening results in the identification of more “micro” medullary cancers, the number of patients with metastases at presentation appears to be decreasing [8, 9, 12].

### Therapy for localized disease

In early or locally advanced MTC, the only curative approach is complete surgical resection of the thyroid and loco-regional lymphadenectomy [1, 3, 13]. Total thyroidectomy rather than unilateral lobectomy is the preferred surgical approach. Up to 10% of patients with sporadic MTC and all patients with inherited MTC have bilateral or multifocal disease [1]; in addition, the latter all have premalignant diffuse C cell hyperplasia. For most patients with MTC confined to the neck and no evidence of involved cervical lymph nodes on preoperative ultrasound, we routinely perform prophylactic bilateral dissection of the central lymph node compartment without prophylactic lateral neck dissection. Prophylactic central neck dissection is not required in patients with small intra-thyroid MTCs with preoperative calcitonin < 20 pg/mL, as metastatic lymph nodes are exceedingly rare in this circumstance [4]. For patients with intraoperative evidence of central cervical lymph node involvement, dissection of the involved lateral neck compartment is also performed. Unfortunately, there is only a 10% cure rate when cervical lymph nodes are involved during initial surgery [1]. Treatment options for patients with persistent or recurrent neck disease include active surveillance, repeat surgery, or other direct therapies (such as radiofrequency ablation, cryoablation, embolization), or systemic therapies. In the treatment choice physician should consider a variety of clinical factors like the tumor volume, the precise location, symptoms, the clinically significant structural disease progression, and the RET mutational status.

### Therapy for advanced disease

Many patients with distant metastases have indolent disease — stable to slow-growing lesions and tumor markers (Ctn and CEA) increasing at a slow pace — that requires active surveillance without the need for systemic therapy for years. Localized treatments like EBRT, surgical resection (metastasectomy), embolization, radiofrequency ablation, or cryoablation are beneficial to control a progressive single focus of metastasis, treat small volume oligometastatic disease when stable in all but one area, alleviate pain, reduce morbidity, or treat refractory diarrhea. EBRT to the neck should be avoided as much as possible because it has not proven to increase the overall survival (OS), causes morbidity to the patient with a decrease in quality of life. Lastly, it increases the risk of fistula formation in the event of a subsequent systemic therapy, particularly with multikinase inhibitors (MKIs) with antiangiogenic properties [14, 15]. Radiation therapy is efficacious in the management of bone metastasis to alleviate pain and to prevent skeletal-related complications (i.e., spinal cord compression, or a pathological fracture) or in the management of a single growing metastatic liver lesion. Bone metastases should also be treated with bisphosphonates or the RANK-L inhibitor denosumab [1, 16–20].

## Systemic therapies

The decision to start systemic treatment should not be taken lightly as it confers toxicities with long-term effects remaining unknown, is not curative, requires long-term use for disease control, and loses efficacy over time due to acquired resistance. In the management of advanced/metastatic patients, the clinician must carefully balance tumor growth rate, quality of life, treatment efficacy, and toxicities [1]. Systemic treatments in patients with advanced MTC should be introduced in the presence of at least one of the following scenarios: [1] progressive (by RECIST) within 12–14 months, [2] symptomatic disease not amenable to any localized or symptom-specific therapies, [3••] tumor invasion to vital structures not amenable to localized therapies, [4] severe, intractable MTC-related diarrhea or paraneoplastic Cushing’s syndrome with lack of an alternative efficacious treatment, and [5] as a relative indication: Ctn or CEA doubling time less than 6 months with small individual lesions that add up to a large tumor burden [3, 10, 14].

### Molecular target agents used as first-line therapy

The categories of drugs that can mainly be used are antiangiogenic MKIs with nonselective RET inhibitors or selective RET inhibitors (Table 1), to date it is not known which is the best sequence of administration of these 2 categories of drugs, the randomized trials in progress will answer this question (Table 2).

**Table 1:** Trials with target therapies in MTC

Drug	Setting	Phase	Patients	mPFS (months)	ORR (%)	DCR (%)	mDOR (months)	mOS (months)	References
Selpercatinib	RET mutant MTC treatment naïve	II	88	NR (24.4-NR)	73 (62–82)	94 (NA)	NR (19.1–NR)	NR	[21••] LIBRETTO-001 trial
	RET mutant – MTC Previously treated	II	55	NR	69 (55–81)	96 (NA)	22.0 (NR–NR)	NR	[21••] LIBRETTO-001 trial
Pralsetinib	RET mutant MTC Treatment naïve	II	21	NR	71 (48–89)	100 (84–100)	NR	NR	[22••] ARROW trial
	RET mutant – MTC Previously treated	II	55	NR	60 (46–73)	98 (90–100)	NR	NR	[22••] ARROW trial
Vandetanib	Unselected advanced MDT	III	231	NR	45 (NA)	87 (NA)	NR (NA)	NR	[23] ZETA trial
Cabozantinib	Unselected advanced MDT	III	219	11.2	28 (NA)	NA	NA	26.6	[24] EXAM trial
Anlotinib	Locally advanced / metastatic MTC	IIB	62	22.4	48.4	88.7	15.6	50.4	[25] NCT02586350
Axitinib	Advanced MTC	CUP	13	9.4	23.1	61.5	na	18.9	[26]
	Advanced MTC	II	6	na	0	83	na	na	[27] NCT00389441
Lenvatinib	Unresectable progressive MTC	II	59	9.0	36	80	NR	16.6	[28] NCT00784303
	Progressing metastatic MTC	II	9	9.2	22	100	na	12.1	[29] NCT01728623
Sorafenib	Advanced sporadic MTC	II	16	17.9	6	94	20.7	NR	[30] NCT00390325
Sunitinib	Metastatic MTC	II	7	na	50	71	na	na	[31] NCT00519896
	Locally advanced / metastatic MTC	II	26	16.5	38.5	88.5	12	29.4	[32] NCT00510640
Pazopanib	Metastatic progressive MTC	II	35	9.4	14.3	71.4	12	19.9	[33] NCT00625846
Vandetanib + Bortezomib	Advanced / metastatic MTC	I	19	na	31.6	78.9	na	na	[34] NCT00923247
90-Y-DOTATOC	Metastatic MTC	II	31	na	29	na	na	15.7	[35]

*CUP* Compassionate use program; *DCR* disease control rate; *mDOR* median duration of response; *mOS* median overall survival; *mPFS* median progression-free survival; *MTC* medullary thyroid cancer; *NA* not available; *NR* not reached; *ORR* objective response rate

**Table 2:** Ongoing trials

Drug	Setting	Phase	Status	References
Selpercatinib vs. cabozantinib vs. vandetanib (LIBRETTO-531)	Progressive, advanced, kinase inhibitor naïve, RET mutant MTC	III	Recruiting	[36••] NCT04211337
Pralsetinib vs. cabozantinib vs. vandetanib (AcceleRET-MTC)	Unresectable, locally advanced, or metastatic, RET mutant MTC	III	Recruiting	NCT04760288
TPX-0046	RET-altered cancers (fusion-mutation)	I/II	Active, not recruiting	NCT04161391
TAS0953/HM06	RET-altered cancers	I/II	Recruiting	NCT04683250
TY-1091	RET-altered cancers	I/II	Recruiting	NCT05675605
SY-5007	RET-altered cancers	I	Recruiting	NCT05278364
BOS172738	RET-altered cancers	I	Active, not recruiting	NCT03780517
Pembrolizumab	Metastatic MTC	II	Recruitment completed	NCT03072160
Nivolumab + Ipilimumab	Metastatic MTC	II	Active, not recruiting	NCT03246958
Regorafenib	Metastatic MTC	II	Recruiting	NCT02657551
Surufatinib	Advanced MTC	II	Recruitment completed	NCT02614495
Anti-CEA x Anti-HSG TF2 bispecific antibody and 68 Ga-IMP-288 Peptide	Recurrent MTC	I/II	Recruitment completed	NCT01730638
Radiolabelled CCK-2/Gastrin receptor analogue	Progressive or metastatic MTC	I	Recruitment completed	NCT03246659
177Lu-PP-F11N	Metastatic MTC	I	Recruiting	NCT02088645
CART-GFRα4 cells	Recurrent or metastatic MTC	I	Recruiting	NCT04877613
HA121-28 tablets	Unresectable locally advanced or metastatic MTC	II	Recruiting	NCT04787328
LBH589	Metastatic MTC	II	Recruitment completed	NCT01013597

*MTC* Medullary thyroid cancer

Vandetanib and cabozantinib are the MKIs currently in use. Vandetanib targets VEGFR, RET, and EGFR, while cabozantinib targets VEGFR 1,2 c-MET, and RET. In a phase II study limited to 30 patients with metastatic or unresectable hereditary MEN2A MTC, 6 (20%) patients achieved a partial response to vandetanib, while 16 (53%) achieved disease stability for at least 24 weeks [37]. An international phase III study randomized 231 patients with sporadic or hereditary MTC to receive vandetanib or placebo. Patients with both progressive and stable disease were eligible for enrollment. After a median follow-up of 24 months, progression-free survival (PFS) was significantly prolonged for patients assigned to vandetanib compared with placebo (hazard ratio [HR] 0.46, 95% confidence interval [CI] 0.31–0.69) [23]. The vandetanib group did not achieve the median PFS but it was expected to be 30.5 months compared with 19.3 months for the placebo group. The objective response rate (ORR) was significantly higher in the vandetanib group (45% vs. 13%). A post hoc analysis of this trial showed that the outcome of patients with progressive was similar to that of stable disease [38]. Of interest, calcitonin levels decreased dramatically after vandetanib therapy, but the marker decrease did not directly correlate with changes in tumor volume; thus, calcitonin may not be a reliable marker of tumor response in patients receiving RET inhibitor therapy [37]. Recently, the activity of vandetanib in MTC not carrying the RET mutation was investigated in depth. Data from 47 patients treated with vandetanib in phase III OBS14778 study were pooled with 50 prospectively and retrospectively enrolled patients with symptomatic, aggressive, sporadic, unresectable, locally advanced/metastatic MTC. Overall, 97 patients were screened and 79 were evaluated for efficacy, of which 58 were RET mutation positive and 21 were RET mutation negative. ORR was 5.0% for RET mutation-negative patients and 41.8% for RET mutation positive patients (NCT01945762). Cabozantinib was investigated in a controlled randomized trial of 219 patients with progressive, metastatic, or unresectable locally advanced MTC. A significant prolongation of PFS was observed for cabozantinib treatment compared with placebo (11.2 vs. 4.0 months; HR 0.28, 95% CI 0.19–0.40). Moreover, PFS was significantly better in the subgroup of patients treated with cabozantinib compared with placebo whose tumors presented RAS mutation [39]. Partial responses were observed in 28% vs. 0%. Median OS improved non-significantly by 5.5 months with cabozantinib therapy (26.6 vs. 21.1 months; HR 0.85, 95% CI 0.64–1.12) [24].

The FDA approved the use of vandetanib or cabozantinib for patients with locally advanced or metastatic MTC who are not eligible for surgery and whose disease is causing symptoms or growing and NCCN guidelines recommend these drugs as first line [10••].

Because of the insufficient activity of vandetanib in patients with no identified RET mutations, recently EMA restricted the vandetanib indication to patients with a RET-positive tumor.

Access to vandetanib is also restricted through a vandetanib risk evaluation and mitigation strategy (REMS) program because of potential cardiac toxicity involving prolongation of the QTc interval [40], while rare adverse events with cabozantinib include severe bleeding and gastrointestinal perforations or fistulas; severe hemorrhage is a contraindication for cabozantinib.

The advent of comprehensive next-generation sequencing (NGS) of tumors, identifying molecular drivers of tumorigenesis, allowed the development of targeted therapies with greater efficacy and less potential off-target adverse events.

Selpercatinib and pralsetinib are the current selective RET inhibitors in use for MTC patients. These drugs were tested in prospective studies recruiting both treatment-naive and pretreated patients with RET-mutated MTC.

NGS is the preferred method to detect RET mutation, if available [3••]. In the single-arm open-label study, LIBRETT0-001, selpercatinib was tested in 143 patients with advanced or metastatic RET-mutated MTC. ORR was 73% in treatment-naïve and 69% in previously treated patients, with a complete response rate of 9% and 11%, and a partial response rate of 60 and 61%, respectively [21••]. After a median follow-up of 16.7 and 11.1 months in the two groups, median PFS was not reached. A randomized phase III trial (NCT04211337), comparing selpercatinib with either cabozantinib or vandetanib, at the physician’s choice, is ongoing. Because of the rapid tumor shrinkage observed with selpercatinib, a neoadjuvant therapy trial (NCT04759911) is recruiting patients with locally advanced primary tumors.

The most reported grade 3 and 4 toxicities of selpercatinib were hypertension (21%), increased alanine aminotransferase (11%), increased aspartate aminotransferase (9%), hyponatremia (8%), and diarrhea (6%).

Pralsetinib activity has been investigated in the single-arm open-label ARROW study. Among the 21 treatment-naive patients, the ORR was 71%, including a 5% complete response rate. In 55 patients previously treated with cabozantinib and/or vandetanib, the ORR was 60% of which only 1.8% were complete responses [22••]. Pralsetinib was generally well-tolerated, with the most common reported grade 3–4 treatment-related adverse events being hypertension (11%) and neutropenia (10%) [41]. Due to these encouraging results, RET mutation analysis should always be done when anticipating the need to start systemic therapy.

Selective RET inhibitor drugs have achieved a higher response rate and a more tolerable side effect profile considering MKI, vandetanib, or cabozantinib (but data supporting the use of pralsetinib-selpercatinib are less robust than in phase III studies of MKIs and ESMO guidelines shows as preferred option vandetanib and cabozantinib). A complete response was achieved in only about one over 10 patients with this specific-RET inhibitor, but these therapies can potentially provide long-term disease stabilization and delay progression in selected patients. However, no studies have yet reported the effects of these agents in improving survival.

**Fig. 1:**
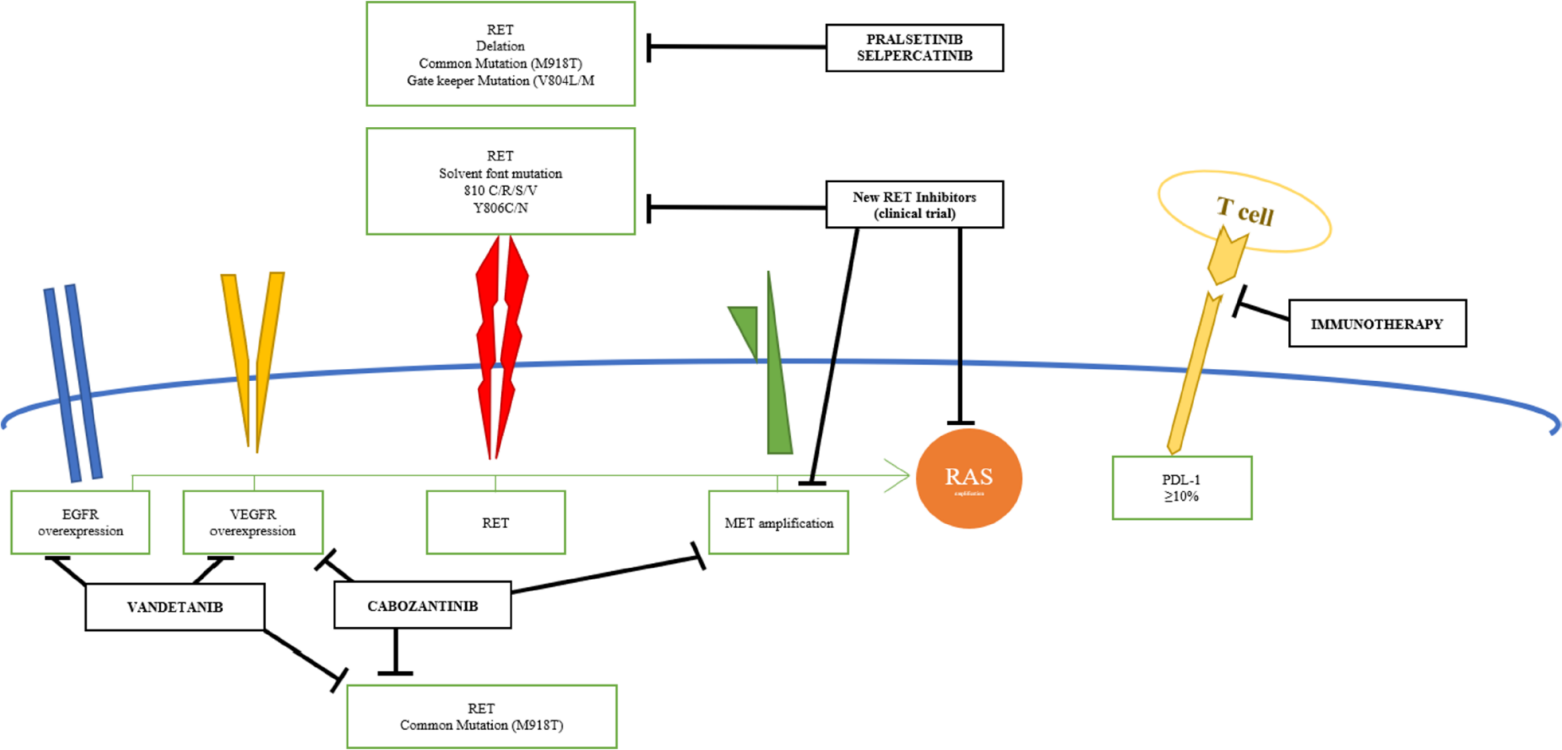
RET- target drugs.

The type of RET mutation may direct the choice of first-line therapy. Selective RET inhibitor drugs, such as selpercatinib and pralsetinib appear to have excellent inhibitory potential against the most common somatic RET M918T mutation and other pathogenic RET mutations or deletions. In addition, these inhibitors can overcome the gatekeeper RET V804L/M mutations, which are known to confer resistance to both cabozantinib and vandetanib by preventing these molecules from inserting into the ATP-binding pocket of the kinase portion of the receptor [23, 24] (Fig. 1).

The results of these studies led to the following regulatory approvals:

- In Europe, selpercatinib has been approved only after vandetanib or cabozanitnib, while in FDA approved regardless of whether or not patients had previously been treated with MKIs. DNA quantitative PCR or NGS are the preferred approaches for testing RET mutation.
- Pralsetinib has been EMA and FDA-approved for RET-mutant advanced or metastatic MTC.

### Subsequent lines

Sorafenib, sunitinib, or lenvatinib are reasonable options for patients who have failed either or both selective and non-selective anti-RET drugs but only if clinical trials are not available or are not appropriate. Lenvatinib was investigated in a phase II study of 59 patients [28]. The best overall response rate was 36% (95% CI 24–49%), all partial responses, while 44% had disease stability. The two cohorts of naive and pretreated patients had similar response rates. Median PFS and OS were 9.0 months (95% CI 7.0-not achieved) and 16.6 months (95% CI 14.0-not achieved), respectively [28]. Limited results demonstrated the activity of sorafenib and sunitinib in MTC. The largest trial with sorafenib included 16 patients. One patient achieved a partial response and the median PFS was nearly 18 months [30]. Partial response (*n* = 3) or durable stable disease (*n* = 3) was also reported in six of eight MTC patients participating in a phase I study of a combination sorafenib and tipifarnib [42]. Sunitinib was investigated in an open-label, phase II trial in 26 patients with progressive refractory MTC with a median follow-up of 25 months, the ORR was 38.5 (22.6–56.4 months), with a median PFS of 16.5 months [32]. Anlotinib is an MKI that inhibits VEGFR, FGFR, PDGFR, and c-Kit. Its activity in MTC was investigated in a randomized, double blind, placebo-controlled phase IIB study (ALTER 01031), enrolling 91 patients 2:1. In the anlotinib arm, median OS was 50.4 months and median PFS reached 22.4 months, with an ORR of 48.4% and 40.3% of stable diseases [25]. The activity of the MKI axitinib was evaluated in a compassionate use program involving 13 MTC patients. Only 3 patients obtained a disease response (all partial) and in 5 cases stability of disease was achieved (ORR 23.1%, DCR 61.5%) [26]. This molecule has been also investigated in a phase II study on advanced thyroid cancer. Six MTC patients were enrolled and none of them obtained a response of disease, while in 5 cases (83%) disease was stable [27]. Pazopanib is a small molecule that inhibits principally VEGFR, PDGFR, and c-Kit. MC057H is a phase II trial that investigated its activity in progressive MTC patients. Among the 35 enrolled individuals, there have been 5 partial responses, with a median duration of response of 1 year, and 20 stabilities of disease. Median PFS and median OS were 9.4 and 19.9 months, respectively. Evaluating potential associations between disease response and tumor markers, it was found that, in this subgroup of patients, CEA reduction significantly relates to longer PFS [33]. Bortezomib is an inhibitor of chymotrypsin-like activity of the 26S proteasome, approved for the treatment of hematologic malignancies. Preclinical studies indicate that this molecule can also reduce RET levels in vitro and inhibit MTC cell lines’ growth. A recent phase I study evaluated the safety and tolerability of the combination of bortezomib with vandetanib. Nineteen MTC patients were enrolled and, although some RECIST responses were achieved (6 patients), the combination therapy was judged not better than single-agent vandetanib with added toxicities, so planned phase II study was not pursued [34].

If the patient progresses on a preferred second-line molecular target therapy, systemic chemotherapy can be administered using dacarbazine or combinations including dacarbazine [43] or as preferred choice, if possible, the inclusion in a clinical trial is to be considered. EBRT can be used for local symptoms while intravenous bisphosphonate therapy or denosumab for bone metastases. Best supportive care is also recommended [10, 44].

**Fig. 2:**
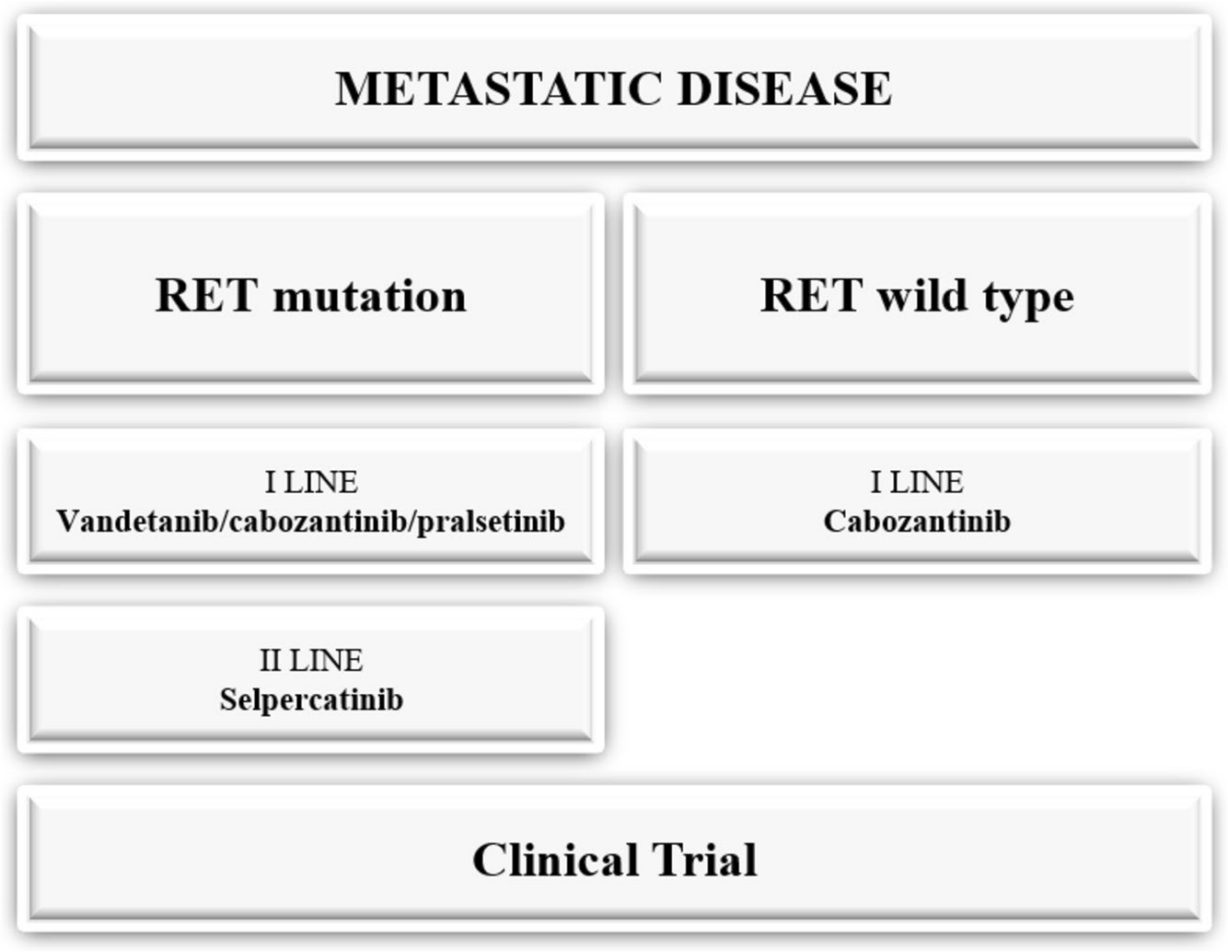
Flowchart treatment for metastatic disease in our Institution.

Similarly to other neuroendocrine tumors, MTC expresses the somatostatin receptor subtype 2. In a single-arm phase II clinical trial, 31 patients with progressive metastatic MTC and with tumor uptake at pre-therapeutic scintigraphy, treatment with radiolabeled octreotide, [90-octrio-DOTA]-TOC, resulted in decreased calcitonin levels in nine patients (29%). The responsive patients had a significantly longer median survival (109 months from the time of diagnosis compared with 80 months for non-responders) [35]. A randomized phase III clinical trial is warranted to confirm these results (Fig. 2).

## Emerging therapies

Emergent RET mutations at the solvent front (G810C/R/S/V) and hinge region (Y806C/N) have been associated with the progression of disease and resistance to selpercatinib and pralsetinib.

Other resistance mechanisms to the selective RET-inhibitors include the development of MET and KRAS amplifications. Even though these resistance mechanisms occur infrequently, it is important to check for the development of new mutations when progression occurs after an initial response to therapy. Real-time identification of emergent mutations in patients who progress on treatments can offer insight into possible resistance mechanisms optimizing further treatment planning [45].

Therefore, precision medicine represents the present/future strategy for MTC as well as many types of cancers. This approach is favored by the accessibility and the wider use of tissue NGS and liquid biopsy. New RET-inhibitor, immunotherapy, radioimmunotherapy, MTC tumor–derived vaccines and drugs interfering with the methylation of DNA could represent the most interesting options for the future.

Several next generation RET-inhibitors are in development [7•] (Table 2). TPX-0046 is currently undergoing a phase I/II clinical trial (NCT04161391) in adult subjects (⩾18 years old) with progressive or advanced-metastatic solid tumors harboring RET fusions or mutations.

TPX-0046 is a novel, potent, and selective inhibitor of both RET and another proto-oncogene: SRC. Unlike other RET inhibitors, this drug presents a rigid macrocyclic structure which makes it active against various mutations, including the solvent front mutation (SFM) RET G810 that conveys resistance to other selective RET inhibitors but not the gatekeeper V804, which limits its effectiveness in some patients with MTC especially whose disease harbor both gatekeeper and SFMs [7•]. BOS172738 is a small molecule RET inhibitor that has demonstrated robust low nanomolar potency (kd ⩽1 nM) against wild-type RET and fusion and mutated protein receptors including M918T, V804L, and V804M, while keeping approximately 300-fold selectivity against VEGFR2. BOS172738 produced durable tumor regression and tumor growth inhibition at similar or lower IC50 concentrations compared with ponatinib in preclinical studies. A phase I (NCT03780517), open-label, multicenter, dose escalation study is currently ongoing to evaluate safety, tolerability, pharmacokinetics, and pharmacodynamics in adult patients (⩾18 young) with advanced solid tumors with RET alterations [7•]. TAS0953/HM06 is a selective RET inhibitor undergoing a phase I/II clinical trial (MARGARET study) (NCT04683250) in adult patients with advanced, progressive, or metastatic RET-altered solid tumors with or without prior MKI therapy [7•].

Immunotherapy represents an option in the USA for patients with MTC with non-druggable mutations with tumor mutational burden higher than 10 [mutations/megabase]. The NCCN guidelines consider Pembrolizumab an option for this specific case, both in the first and subsequent line [10••]. Recently several studies evaluated the expression of the immune co-inhibitory receptors PD-1, CTLA-4, TIM-3, LAG-3, and TIGIT and had identified MTC as a more immunogenic tumor, or at least not so immunologically “cold” as previously reported in small sample studies [46–49]. Very preliminary results of a phase II trial evaluating nivolumab plus ipilimumab in patients with aggressive thyroid cancer (NCT03246958) are available. Indeed, 7 patients with progressive MTC and prior MKI failure were included in an exploratory cohort of the study and assessed for radiographic response based on RECIST v1.1 criteria. A lack of partial response is reported for all the 7 patients [50]. One of the possible approaches of immunotherapy is to induce host immunity against the tumor by administering tumor-derived vaccines or inoculations of transfectant tumor cells that express specific cytokines. Some promising results have been obtained with stimulated dendritic cells. In a small study of only 7 patients, dendritic cells obtained from each patient's tumor were stimulated in the presence of calcitonin and CEA antigen [51]. Following periodic administrations, one patient achieved a partial response, including complete regression of liver metastases, associated with a 70% reduction in serum tumor markers. Two other patients had mixed responses. In another study of 10 patients, dendritic cells were stimulated with lysates of each patient's primary tumor [52]. Given the peculiar overexpression of CEA on MTC cells, radiolabeled anti-CEA monoclonal antibodies have been studied for radioimmunotherapy. In particular, a bispecific recombinant anti-CEA/anti-ethylenetriamine pentaacetic acid (DTPA)-indium (BsMAb) antibody, followed four days later by a bivalent 131I–labeled hapten, demonstrated some activity in a small cohort of patients [53]. In a subsequent single-arm study in patients with progressive metastatic MTC (defined as calcitonin doubling time of less than two years), the median OS after administration of this therapy was 110 months [45]. This compares favorably with the median survival of a contemporary untreated cohort of only 60 months.

Recently, proteome-based stratification of 102 MTCs revealed three molecularly heterogeneous subtypes that are distinct in genetic drivers, epigenetic modification profiles, clinicopathologic factors, and clinical outcomes that in the future could represent a guide for choosing the more appropriate treatment. Cluster III, “Mesenchymal” (32% of the MTC considered), revealed a relatively higher level of DNA methylation besides enrichment of RETM918T mutation and STAT3 signaling activation, possibly a cause of severe prognosis in mesenchymal tumors [54•]. On the other hand, the higher level of DNA methylation could be targeted since numerous small molecules that target the epigenetic regulatory enzymes have been identified recently, some of which show promise as anticancer treatments. Indeed, several epigenetic drugs have been approved by the FDA and are commercially available for the treatment of hematologic tumors, such as DNA methyltransferase inhibitor (DNMT) inhibitors (azacytidine and decitabine), histone deacetylase inhibitor (HDAC) inhibitors (vorinostat, romidepsin, belinostat, panobinostat, and chidamide), enhancer of zeste homolog (EZH2) inhibitor (Tazemetostat) [55••]. Additionally, there are several clinical trials involving inhibitors of epigenetic regulators that are ongoing and that could synergize if combinate with small molecules targeting chromatin or immunotherapy providing additional opportunities for their future clinical application [56].

## Conclusion

Metastatic MTC is a rare disease with a potentially severe prognosis. Even though many drugs were recently approved for MTC, we do not have either international shared nor standard options after the second line. Tumor genetic profiling and ongoing trials will help clinicians to better understand the more appropriate therapy and the best treatment sequence.
